# Clarifying relationships between cranial form and function in tapirs, with implications for the dietary ecology of early hominins

**DOI:** 10.1038/s41598-020-65586-w

**Published:** 2020-06-01

**Authors:** Larisa R. G. DeSantis, Alana C. Sharp, Blaine W. Schubert, Matthew W. Colbert, Steven C. Wallace, Frederick E. Grine

**Affiliations:** 1 Department of Biological Sciences, Vanderbilt University, Nashville, Tennessee 37235-1634 USA; 2 Department of Earth and Environmental Sciences, Vanderbilt University, Nashville, Tennessee 37235-1805 USA; 3 Institute of Lifecourse and Medical Sciences, University of Liverpool, Liverpool, L69 3BX United Kingdom; 4 School of Science and Technology, University of New England, Armidale, New South Wales 2351 Australia; 5 Department of Geosciences and Don Sundquist Center of Excellence in Paleontology, East Tennessee State University, Johnson City, Tennessee 37614 USA; 6 Department of Geological Sciences, The University of Texas at Austin, Austin, Texas 78712 USA; 7 Department of Anthropology, Stony Brook University, Stony Brook, New York 11794 USA; 8 Department of Anatomical Sciences, Renaissance School of Medicine, Stony Brook University, Stony Brook, New York 11794 USA

**Keywords:** Zoology, Anatomy, Ecology, Evolutionary ecology, Palaeoecology, Evolution, Anthropology, Palaeontology

## Abstract

Paleontologists and paleoanthropologists have long debated relationships between cranial morphology and diet in a broad diversity of organisms. While the presence of larger temporalis muscle attachment area (via the presence of sagittal crests) in carnivorans is correlated with durophagy (i.e. hard-object feeding), many primates with similar morphologies consume an array of tough and hard foods—complicating dietary inferences of early hominins. We posit that tapirs, large herbivorous mammals showing variable sagittal crest development across species, are ideal models for examining correlations between textural properties of food and sagittal crest morphology. Here, we integrate dietary data, dental microwear texture analysis, and finite element analysis to clarify the functional significance of the sagittal crest in tapirs. Most notably, pronounced sagittal crests are negatively correlated with hard-object feeding in extant, and several extinct, tapirs and can actually increase stress and strain energy. Collectively, these data suggest that musculature associated with pronounced sagittal crests—and accompanied increases in muscle volume—assists with the processing of tough food items in tapirs and may yield similar benefits in other mammals including early hominins.

## Introduction

Discerning the form–function relationship between skulls, teeth and diet is paramount to our understanding of ecology and evolution. The majority of paleoecological interpretations of Cenozoic mammals rests on the assumption that one can potentially infer the diets of extinct taxa by comparing their anatomical features to those of living species with known diets. Some relationships between craniodental form and function are well-established, such as the advent of high crowned and more molariform cheek teeth together with broadened muzzles that accompanied increased grazing in horses and their relatives^1,2^. Similarly, there is good evidence among primates that the mechanical properties of food items may impact skeletal and dental morphologies related to their ingestion and mastication^3–5^. As a result, anatomical attributes have been widely used in attempts to infer the dietary habits of extinct hominin species (e.g.^6–13^). However, the success that these biomechanical inferences have had for the retrodiction of hominin diets has been a matter of discussion^14–19^.

Much of the debate concerning the dietary habits of extinct hominins has focused on *Paranthropus* and, in particular, on the East African species *P. boisei*. Members of this genus are characterized by enlarged postcanine dentitions, cheek teeth with very thick enamel, dished faces in which the cheeks protrude anterior to the nasal aperture resulting in anteriorly placed masseteric muscle attachments, and sagittal and nuchal crests that are more pronounced, indicating enlarged temporalis muscles in comparison to species of *Australopithecus* such as *A. afarensis* and *A. africanus*^20^. These features, which bespeak an enhanced masticatory system have consistently been interpreted as adaptations to and testaments of hard-object feeding (e.g.^7–9,13,21^). However, the models that have been employed have not been without criticism^14,16,17,22–25^.

The microwear and isotopic signatures of *P. boisei* are inconsistent with the consumption of hard-foods^26–28^. Diet is not the same thing as dietary adaptation. As noted by Lee-Thorp^29^, these data should have portended the demise of the popular moniker—“Nutcracker Man” —for *P. boisei*. Nevertheless, the adaptationist paradigm persists^9,21^ despite the fact that both load magnitude and load frequency are implicated in osseous metabolic activity that results in alterations to bone mass and architecture^30–32^ and experimental evidence that hard object feeding, whether habitual or on an occasional “fallback” basis, does not explain the dentognathic morphology of *P. boisei*^33^.

There continues to be profound disagreement between morphological interpretations and dietary proxy data regarding the dietary ecology and functional significance of a suite of craniodental features of *Paranthropus*. Interpreting the dietary ecology and functional significance of the robust craniodental features of *P. boisei* is complicated by the lack of any extant primate analogues with a similar suite of features^34^.

As a well-studied group, carnivorous mammals, specifically members of the order Carnivora, are typically used as model organisms for interpreting the function of pronounced sagittal crests and large temporalis muscles, and in particular, are used to correlate these features with high bite forces and hard-object feeding (e.g.^35^). As expected, carnivorans with some of the largest and most pronounced sagittal crests, engage in durophagy and are capable of cracking bones open^35^; most notably—the spotted hyena, *Crocuta crocuta*. Finite element analysis (FEA) of *C. crocuta* demonstrates their ability to withstand increased stress from high bite forces^36^. Dental microwear also documents bone-cracking behavior in hyenas^37–40^, demonstrating hard-object feeding in all three species of extant hyenas while documenting softer food consumption (i.e., lower complexity values) in cheetahs with similar carnassial teeth^39^. Interestingly, the herbivorous giant panda (a member of Carnivora, *Ailuropoda melanoleuca*) also has a large sagittal crest^41–43^ yet has dental microwear indicative of tough and hard food items (see Materials and Methods for descriptions of DMTA attributes)^44^. This brings into question the ability to infer a correlation between crest morphology and diet, specifically for hard-object feeding^45^. While the examination of great apes with pronounced sagittal crests (e.g. gorillas and orangutans) may be revealing, no primate comparisons are perfect—many great apes consume hard and tough foods and the presence of sagittal crests is a sexually dimorphic trait, complicating understandings of morphological forms and functions^13,17^.

We suggest that tapirs (*Tapirus*; Tapiridae; Perissodactyla), being large herbivorous mammals that evince variable sagittal crest development among congeners, are a potential model for examining the correlation between the textural properties of food and morphology related to enhanced masticatory musculature. The sagittal crest of extant New World tapirs varies from a prominent, but low sagittal crest of less than 1 cm height in *Tapirus pinchaque* (the mountain tapir), a taller (greater than 4 cm in adults) and laterally compressed sagittal crest in *Tapirus terrestris* (the lowland, South American, or Brazilian tapir), and having a sagittal table rather than a sagittal crest in *Tapirus bairdii* (Baird’s tapir)^46^. Further, all tapirs are herbivorous and have similar tooth morphologies (low crowned and bilophodont teeth; enamel thickness is also similar among tapirs, with *T. terrestris* intermediate between *T. bairdii* and *T. pinchaque*; see supplemental methods, supplemental results, Supplemental Fig. 1 and Supplemental Table 1)^47^, yet they are known to eat variable degrees of tough leaves, fruits, hard seeds, and other foliage^48–54^). Specifically, *T. bairdii* is known to predate large seeds^48–50^, while *T. terrestris* and *T. pinchaque* consume a higher incidence of foliage (soft to tough foods)^48–54^. Extinct tapirs from the Pleistocene of North America that may also provide insight include: *Tapirus haysii*, *T. lundeliusi*, and *T. veroensis*. The closely related *T. veroensis* and *T. haysii* have lower sagittal crests (i.e. adult height is less than 2 mm)^55,56^ than the extinct *T. lundeliusi*, and extant *T. pinchaque* and *T. terrestris*. Only one fossil tapirid (even when compared to all tapirs including Old World tapirs), the dwarf tapir *Tapirus polkensis*, is characterized as having low parasagittal ridges that do not unite to form a sagittal crest ~75% of the time, with the other ~25% of adults having a sagittal crest when it occurs at the late Miocene to early Pliocene Gray Fossil Site (GFS) in Tennessee^57^. Previous work^58^ examined *T. polkensis* from the GFS, and extant tapirs from throughout the Americas to assess if age (as inferred from tooth eruption) or sexual dimorphism (in extant specimens with known sex data) played a role in sagittal crest formation, but data were equivocal. Further, sagittal crest morphology in *Tapirus* is not correlated with size, with *T. terrestris* being intermediate in size, but with the tallest crest^57^.

Understanding relationships between form, function, and diet requires a multi-proxy approach. FEA is capable of clarifying the potential for extant and extinct taxa to consume particular food types, i.e. their function, as inferred from the mechanical performance of the cranium—removing effects of size^59–63^. In contrast, dental microwear texture analysis (DMTA) has the potential to reveal the textural properties of food consumed during the past few days to weeks of an animal’s life^64,65^, and can clarify the dietary ecology of numerous extinct taxa; as compared to extant taxa with known diets (e.g.^66–68^). Here, we assess the functional significance of sagittal crest morphology as it relates to diet in several extant and extinct tapirs that exhibit a broad range of morphologies. In addition, we integrate FEA and DMTA to clarify the degree to which hard-object feeding is related to sagittal crest morphology in herbivorous tapirs. While relationships between DMTA, observed diet, and form are assessed, we also included extinct tapirs and associated DMTA data to increase the range of forms represented (including *T. polkensis*, which includes forms both with and without sagittal crests). These data have the potential to alter our understanding of relationships between the form and function of cranial morphology, including our understanding of the dietary ecology of early hominins, most notably *P. boisei*.

## Results

### Finite element analysis

**Figure 1:**
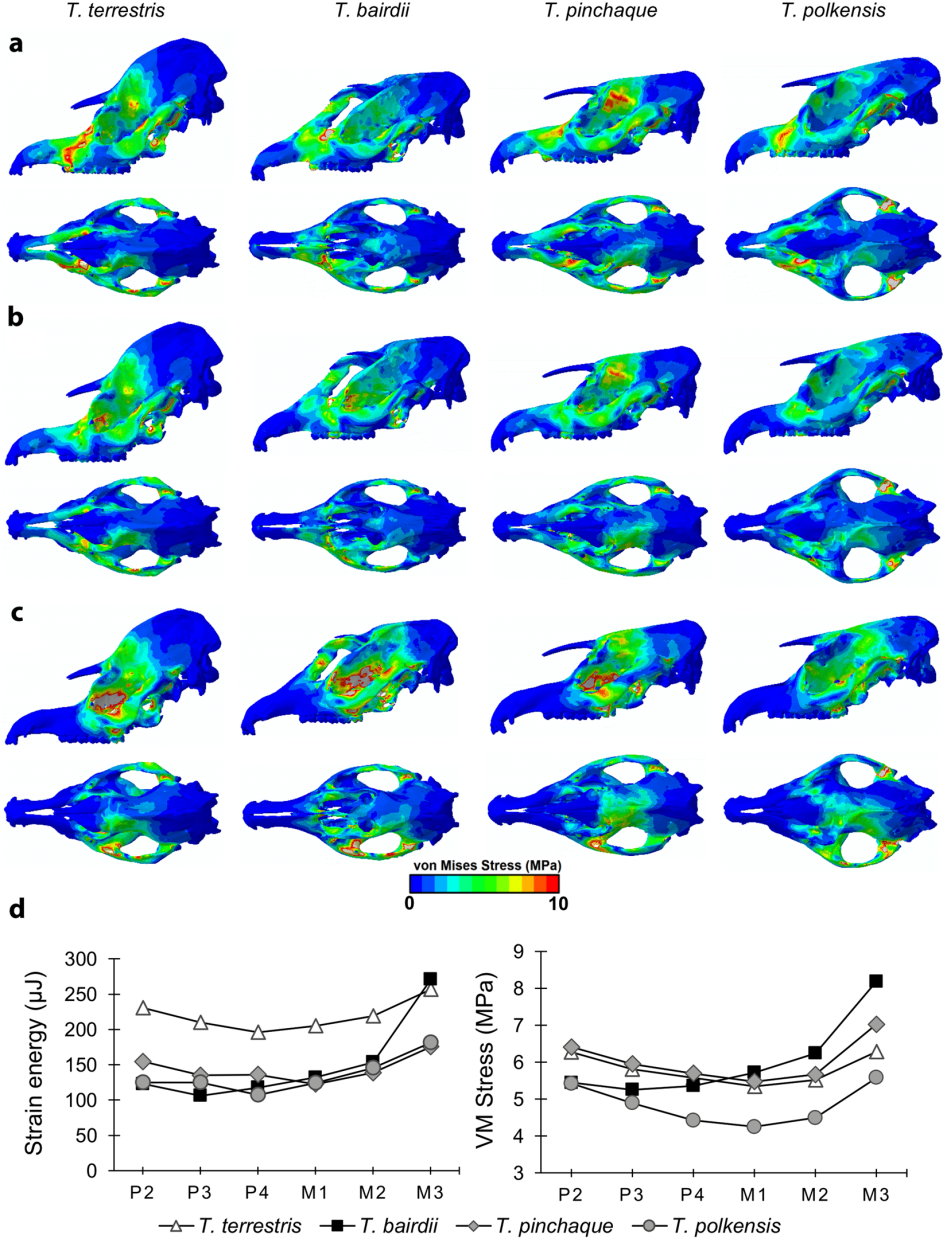
Results from finite element analysis. Predicted distribution of von Mises (VM) stresses across the cranial models of *T. terrestris*, *T. bairdii*, *T. pinchaque* and *T. polkensis* during unilateral biting at the second premolar (**a**), fourth premolar (**b**) and third molar (**c**) shown in lateral (top) and dorsal (bottom) views (warm colors indicate areas of high VM stress and cool colors indicate low stress; grey areas indicate VM stress that exceeds the specified maximum of 10 MPa). Strain energy and adjusted maximum VM stress (**d**) are also shown during unilateral biting at each bite point. Images (**a**–**c**) were produced using Abaqus CAE v6.14 (Simulia) software, based on CT data processed via Avizo v9.0 (FEI, part of Thermo Fisher Scientific) software.

Finite element analysis data are illustrated in Fig. 1, and summarized in Supplemental Tables 2–4. Figure 1 shows the von Mises (VM) stress distribution of each model during unilateral biting at the second and fourth premolar, and third molar. Highest stress was recorded on the biting side around the bite location and the anterior aspect of the orbit for all models. Distribution of stress shows that the peak moves posteriorly so that when biting at the third molar there is minimal stress on the premaxilla.

The “adjusted” maximum stress (maximum stress after removing the top 2% of stress values to account for high stresses caused by point constraints) and strain energy are also shown in Fig. 1 for each bite point. Mean element stress, adjusted maximum stress, strain energy and mechanical efficiency for each model during unilateral biting at each tooth on the left side are shown in Supplemental Table 3, and for bilateral biting in Supplemental Table 4. As the bite point moves posteriorly along the tooth row, stress and strain generally decrease until the middle of the tooth row at approximately the first molar, where the stress and strain start increasing (Fig. 1d). Stress and strain in *T. bairdii*, however, increases along the tooth row so that both stress and strain are highest at the third molar (Fig. 1d, Supplemental Table 3). Performance varied between unilateral biting and bilateral biting so that some species performed better under one compared to the other (Supplemental Tables 3 and 4). Adjusted maximum stress (a proxy for strength) was lowest in *T. bairdii* for bilateral biting and *T. polkensis* for unilateral biting at all bite points, indicating that *T. bairdii* performed better in bilateral biting and *T. polkensis* performed better for unilateral biting (Fig. 1, Supplemental Tables 3 and 4). Maximum stress were highest in *T. polkensis* for bilateral biting at all bite points (Supplemental Table 4), further indicating that the *T. polkensis* model performed better under unilateral biting compared to bilateral biting. Mechanical efficiency increases in all models as the bite point moves posteriorly along the tooth row (Supplemental Tables 3 and 4).

Strain energy (a proxy for work efficiency, or the stiffness of the structure) also varied along the tooth row for each model, with the lowest strain energy generally in the center of the tooth row (Fig. 1). However, the *T. bairdii* model was stiffest and most efficient at the front of the tooth row, likely because of the extra stiffness provided by the ossified nasal septum, and less efficient at M3. The most compliant, and therefore least efficient model, was *T. terrestris*, indicating that it expends more energy on deformation. This species also has the highest and most developed sagittal crest. Mechanical efficiency, a measure of the efficiency at which muscle force is translated to bite force, was highest in *T. bairdii* and lowest in *T. pinchaque* at all bite locations during bilateral and unilateral biting.

### Dental microwear texture analysis

**Figure 2:**
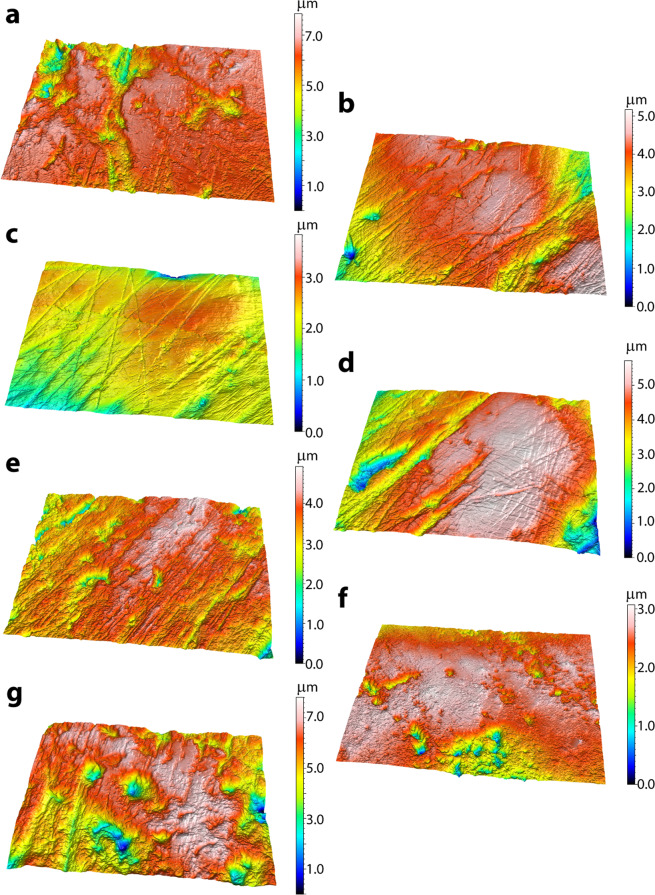
3D surface renderings for extant (**a**–**c**) and extinct tapirs (**d**–**g**). Three-dimensional surface renderings of the following museum specimens are included: *Tapirus bairdii* (**a**, FMNH 34665), *T. terrestris* (**b**, FMNH 34264), *T. pinchaque* (**c**, FMNH 70557), *T. polkensis* (**d**, ETMNH 6820), *T. hasyii* (**e**, UF 89533), *T. lundeliusi* (**f**, UF 224674), and *T. veroensis* (**g**, UF 210890). All surface renderings (**a**–**g**) were produced via SensoMAP software (Sensofar).

**Figure 3:**
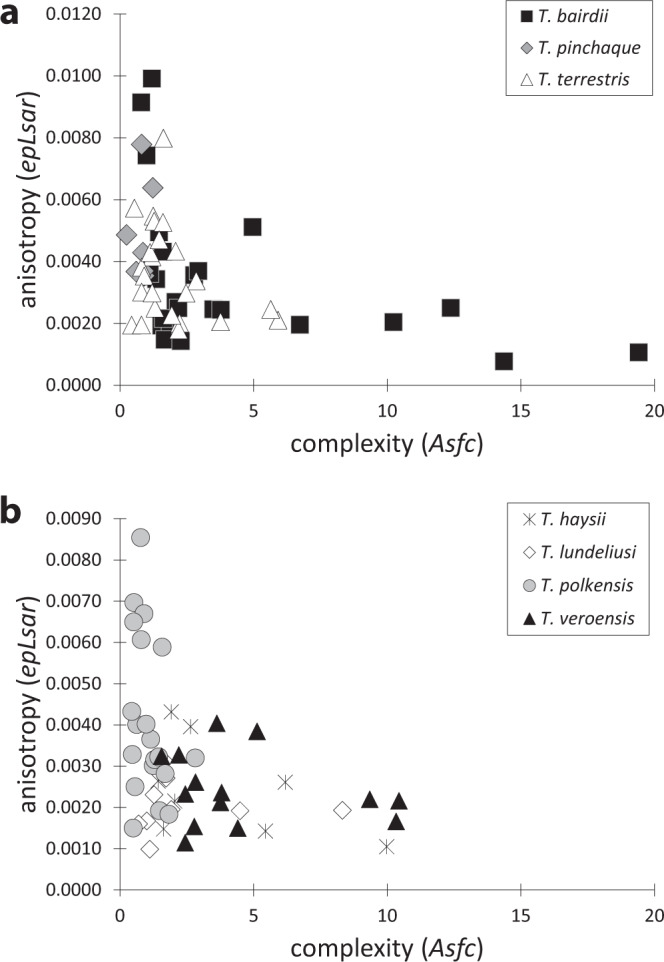
Scatterplots of dental microwear texture complexity (*Asfc*) and anisotropy (*epLsar*) for all extant (**a**) and extinct (**b**) tapirs examined.

Dental microwear textural data are illustrated in Figs. 2 and 3 and summarized in Table 1 and Supplemental Tables 5 and 6 (individual specimen data are included in Supplemental Table 7). Complexity of *T. bairdii* is significantly greater than *T. terrestris* and *T. pinchaque*, and *T. terrestris* is also significantly greater than *T. pinchaque* (Table 1). *T. bairdii* also has significantly greater *Tfv* than *T. terrestris* (Table 1). Anisotropy of *T. pinchaque* is significantly greater than *T. bairdii*, yet indistinguishable from *T. terrestris* (Table 1). None of the extant tapirs can be distinguished using either *HAsfc* metric (3×3 or 9×9); this metric is therefore not discussed further or used to compare with extinct taxa.

**Table 1:** Statistical results for taxonomic comparisons of complexity (*Asfc*), anisotropy (*epLsar*), and textural fill volume (*Tfv*) values for all extant and extinct tapirs here examined.

Variable		*T. pinchaque*	*T. terrestris*	*T. haysii*^*†*^	*T. polkensis*^*†*^	*T. lundeliusi*^*†*^	*T. veroensis*^*†*^
complexity (Asfc)	*T. bairdii*	**<0.001**	**0.036**	0.568	**<0.0001**	0.236	0.106
	*T. pinchaque*		**0.025**	**<0.001**	0.467	**0.029**	**<0.0001**
	*T. terrestris*			**0.040**	**0.034**	0.718	**<0.001**
	*T. haysii*^†^				**<0.001**	0.152	0.484
	*T. polkensis*^†^					0.051	**<0.0001**
	*T. lundeliusi*^†^						**0.018**
anisotropy (epLsar)	*T. bairdii*	**0.015**	0.287	0.319	0.073	**0.047**	0.252
	*T. pinchaque*		0.087	**0.005**	0.254	**<0.001**	**0.002**
	*T. terrestris*			0.080	0.438	**0.006**	**0.039**
	*T. haysii*^†^				**0.023**	**0.001**	0.961
	*T. polkensis*^†^					**0.001**	**0.008**
	*T. lundeliusi*^†^						0.361
textural fill volume (Tfv)	*T. bairdii*	0.096	**0.002**	0.732	**0.015**	**0.033**	0.176
	*T. pinchaque*		0.646	0.098	0.963	0.818	**0.011**
	*T. terrestris*			**<0.01**	0.558	0.835	**<0.0001**
	*T. haysii*^†^				**0.036**	**0.046**	0.477
	*T. polkensis*^†^					0.554	**<0.001**
	*T. lundeliusi*^†^						**0.003**

*p-values <0.05, also in bold. ^†^Denotes extinct taxon.

When extinct and extant tapirs are compared, *T. polkensis* from the GFS are distinctly different from North American Pleistocene tapirs (*T. haysii*, *T. lundeliusi*, and *T. veroensis*) in nearly all DMTA attribute comparisons (with lower *Asfc*, higher *epLsar*, and lower *Tfv* values); with an exception, *T. polkensis* is not significantly lower in *Asfc* or *Tfv* than *T. lundeliusi* (Figs. 2 and 3; Table 1). *T. polkensis* is most similar to *T. pinchaque* (indistinguishable in *Asfc*, *epLsar* and *Tfv*) and *T. terrestris* (indistinguishable in *epLsar* and *Tfv*), whereas *T. polkensis* has significantly lower *Asfc* and *Tfv* than *T. bairdii* (Table 1). The now extinct Pleistocene tapirs here examined are indistinguishable from one another in all DMTA attributes, with the exception that *T. lundeliusi* has significantly lower *Asfc* than *T. veroensis* and significantly lower *Tfv* than both *T. haysii* and *T. veroensis* (Table 1). All Pleistocene tapirs are indistinguishable from *T. bairdii* in *Asfc*, *epLsar*, and *Tfv*, with the exception of *T. lundeliusi* having lower *epLsar* and *Tfv* than *T. bairdii* (Table 1). Further, Pleistocene tapirs are significantly different from the extant *T. pinchaque* and *T. terrestris* in most attributes, with a few exceptions noted in Table 1 (i.e. *T. lundeliusi* and *T. haysii* are indistinguishable from *T. terrestris* in *Asfc* and *epLsar*, respectively; *T. haysii* cannot be distinguished from *T. pinchaque* with *Tfv*; and *T. lundeliusi* is indistinguishable from both *T. pinchaque* and *T. terrestris* with *Tfv*).

Despite variable sagittal crest morphology in specimens of GFS *T. polkensis*, there were no apparent relationships between DMTA attributes and sagittal crest development (the presence or absence of a sagittal crest in adult specimens; noted in Supplemental Table 7, although adult specimens with preserved crania and dental microwear were limited).

## Discussion

FEA results suggest that the presence of a sagittal crest can affect both the energy efficiency (the amount of deformation) and strength (measured by stress) of the cranium in tapirs as it does in carnivorans^36,60,69^. The tapir with the most pronounced sagittal crest (*T. terrestris*) has the highest strain energy (low energy efficiency) and therefore more readily deforms. *T. terrestris* consumes seeds and fruits, potentially more fruits than *T. bairdii* based on stomach contents^50–52^, but isotopic evidence suggests that in rainforests they may be more folivorous (as suggested by ref. ^53^ based on a significant correlation between carbon and oxygen isotope values of tooth enamel, of many of the same specimens examined here for DMTA, linking leaf water and canopy density which is likely only possible if primarily consuming leaf material). A pronounced sagittal crest may confer benefits related to increased temporalis muscle volume—allowing for prolonged mastication which is particularly beneficial when consuming tough-food items with lower nutritional value. However, the presence or development of a sagittal crest (and accompanied larger temporalis muscles) does not appear to be a prerequisite for hard-object feeding as a softer and tougher diet for *T. terrestris* is supported by DMTA. DMTA further suggests that *T. terrestris* consumes softer food items than its congener, *T. bairdii* (based on significantly lower *Asfc* values).

The presence of a sagittal table instead of a sagittal crest in *T. bairdii* results in lower stress and lower strain energy, and coincides with hard-object feeding revealed by DMTA. *T. bairdii* typically occurs in drier forests than *T. terrestris* and is known to predate large seeds (from 20–30 mm), including those of *Manilkara zapota* (commonly known as the sapodilla)^48–50^. In the Calakmul region of Mexico only 53% of dung piles contained intact seeds (noted in ref. ^58^), suggesting that *T. bairdii* is in fact extracting nutrients from these seeds during mastication. Thus, ecological studies, FEA and DMTA all suggest that *T. bairdii* is both capable of and actually consumes hard-objects, e.g. seeds. Interestingly, the protruding and ossified nasal septum may have further improved their ability to consume hard-objects and it may be partly responsible for lower overall stress and strain energy, particularly when biting at anterior teeth, by reducing dorsoventral bending.

FEA and DMTA data of extinct tapirs are variable with some showing evidence of hard-object feeding, and others not. Data from FEA of *T. polkensis* suggests it has a strong skull when modeling unilateral biting but weak for bilateral biting. DMTA suggests a highly folivorous diet and the absence of significant hard-object feeding, despite hickory nuts being found in the stomach region of these fossil specimens and the species yielding highly variable sagittal crest morphology^58^. However, FEA suggests the potential ability to consume hard-objects during unilateral biting. Other extinct Pleistocene tapirs (e.g., *T. haysii*, *T. lundeliusi*, and *T. veronesis*) have sagittal crests (with *T. haysii* and *T. veroensis* having reduced sagittal crests less than 2 mm in height)^55,56^, and primarily consume hard-objects (as evinced by indistinguishable *Asfc* values from *T. bairdii*)—although, *T. lundeliusi* is also indistinguishable from *T. terrestris*. While FEA was not possible on these taxa, a reduced sagittal crest does correlate with hard-object feeding (via DMTA) in extinct taxa, suggesting that reduced sagittal crests in extinct taxa and the absence of sagittal crests in extant *T. bairdii* may have been better able to handle higher stress loads than taxa with more pronounced sagittal crests—although further work is needed.

Collectively, FEA, DMTA, and ecological data suggest that the presence of moderate to pronounced sagittal crests in extant tapirs does not correlate with hard-object feeding, but rather with processing of tough and/or less nutritious vegetative matter—including more folivorous diets. FEA and DMTA largely agree, with the extant tapirs exhibiting sagittal crests (both *T. pinchaque* and *T. terrestris*) eating tougher and softer foods, whereas *T. bairdii* is both more capable of eating harder foods (low stress and strain) and exhibits this behavior as evinced by DMTA (done here) and observed dietary behavior^48–54^. Therefore, sagittal crest morphology in herbivores does not equate to hard-object feeding; in fact, the opposite is true in extant tapirs. Examination of fossil tapirs suggests that relationships between diet and morphology are not exact; tapirs with reduced sagittal crests or sagittal tables tend to consume harder foods—with the notable exception of the variable *T. polkensis* which exemplifies both morphotypes. These data suggest that sagittal crests in herbivorous tapirs may confer different functional advantages than in carnivorans—a sagittal crest allows attachment for larger temporalis muscles in both taxa, providing higher bite force in bone-cracking carnivorans and allowing for increased masticatory processing of tough food in tapirs.

Our combined FEA and DMTA analyses shed light on the functional significance of the sagittal crest and have broad implications for how we interpret mammalian ecology, including that of hominins. For over sixty years anthropologists have investigated and debated the diets of the “gracile” and “robust” australopithecines, the latter including *P. boisei*^70^. Despite an early study which suggested that the presence of sagittal crests in ancient hominins is related to allometry (i.e. with increased body size a sagittal crest provides increased muscle attachment area, if brain size does not increase)^71,72^, the vast majority of studies to date suggest that the disparate craniodental morphology of the “gracile” and “robust” forms stems from dietary differences—although what these diets are is disputed^9,14,21,26–28,59,64,73–76^. Sagittal crest formation is often noted to occur in mammals with diets that require the consumption of either very hard or tough foods (e.g. hyenas, giant pandas) which require increased muscle volume and muscle attachment area^35–39,41–44,48–54^. In primates, the apes with sagittal crests (i.e. gorillas and orangutans) are also known to consume very tough and/or hard food items, and recent studies have demonstrated relationships between the properties of food (e.g. fracture toughness) and jaw muscle activity/jaw robusticity—which subsequently requires increased muscle mass and a sagittal crest^77,78^. From our analysis of tapirs, sagittal crests and associated craniodental morphologies may confer benefits by allowing for prolonged chewing of tough and/or less nutritious foliage, while the tapirs eating hard food items either lack or have less pronounced sagittal crests relative to body size (than *T. terrestris*, for example).

In contrast to extant tapirs which exhibit disparate diets and morphologies, the congeners *P. boisei* and *P. robustus* exhibit similar morphologies yet proxy evidence suggests different diets. Specifically, the robust masticatory morphology of *Paranthropus*^59,64,73,79^ may have conferred multiple advantages in *P. boisei* and *P. robustus*, with the former able to process tougher and/or less nutritious herbaceous matter (potentially for a prolonged period of time) while the latter was capable of eating harder food items. Alternatively, the ability to consume both tough and hard foods for prolonged periods may also have been possible^59^. While morphological and FEA results suggest that *P. boisei* was capable of eating hard-objects^9,59^ (much like *P. robustus*), dental microwear, stable isotope, and plant biomarker evidence point to a diet that may have been dominated by softer and/or more abrasive foods (e.g. grasses, ferns, sedges, and aquatic food sources)—likely specializing on tough C_4_ grasses^10–12^, 6826^28,75,80^. Much like late Cenozoic horses with high-crowned teeth, which were capable of eating abrasive grasses but many times consumed a mixture of browse and grass^1,2^, potential and realized diets are not always in agreement. Most notably, and as revealed here, relationships between form, function, and diet are complex and require multiple lines of evidence and a diverse suite of extant analogs. Further, herbivorous analogues such as tapirs are important models for inferring dietary relationships as revealed by morphology and suggest that pronounced sagittal crests can be advantageous and correlated with softer/tougher diets in mammalian herbivores.

## Materials and Methods

### Finite element analysis

Crania and mandibles representing four species of tapirs were CT scanned at the University of Texas High-Resolution X-ray CT Facility (UTCT) in Austin, Texas. The scanned material was provided by the following institutions: the Texas Memorial Museum (TMM), the American Museum of Natural History (AMNH), the Museum of Vertebrate Zoology, Berkeley (MVZ), and the East Tennessee State University and General Shale Brick Natural History Museum and Visitor Center (ETMNH). Specimens used in the finite element analysis include:

#### Tapirus terrestris

TMM M-16; San Antonio Zoo; scanned with an interslice spacing of 1.0 mm, and an interpixel spacing of 0.5918 mm.

#### Tapirus bairdii

AMNH 80076; Honduras, near Tela; scanned with an interslice spacing of 1.0 mm, and an interpixel spacing of 0.5468 mm.

#### Tapirus pinchaque

MVZ 124091; Rio San Jose, 2700 m, Moscapan, Huila, Colombia; scanned with an interslice spacing of 0.7 mm, and an interpixel spacing of 0.2197 mm.

#### Tapirus polkensis

ETMNH 3519; Gray Fossil Site; voxel size = 0.1694 mm. This specimen represents one of the ~25% of specimens which have a single sagittal crest.

CT data were processed in Avizo v9.0 (FEI, part of Thermo Fisher Scientific), where the cranium was separated from the mandible and 3D surface reconstructions were generated. The surface meshes were then cleaned and converted to solid volume meshes composed of 4-node tetrahedral elements of between 1.3 million and 2.1 million elements (see Supplemental Table 2) and exported as Abaqus input files (*.inp) for analysis in Abaqus CAE v6.14 (Simulia).

Muscle force estimates where produced using the dry skull method^81^ from digital models in their original size, and applied to the skulls by distributing the load over the temporalis, masseter, and pterygoid muscle origins. Muscle orientations were determined by creating a local coordinate system between the origin and the corresponding insertion on the mandible. Each model was constrained by a single node at both temporomandibular joints (TMJ) and at each bite point for unilateral and bilateral biting at each premolar (P2-P4) and molar (M1-M3). For bilateral biting, maximum muscle contraction was simulated on both sides, whereas for unilateral biting, the balancing-side (non-biting side) was adjusted to 60% of the maximum muscle force. The left TMJ was fully constrained against translation in any axis, the right TMJ was constrained in the y- and z- axis to allow lateral displacement of the skull, and the bite point(s) were constrained in only the axis perpendicular to the occlusal plane. All models were assigned as homogeneous and isotropic with average values of Young’s modulus (E = 20 GPa) and Poisson’s ratio (ν = 0.3) for mammalian bone^82^, and all analyses were linear and static.

Due to the variation in size among the tapir species, the models were standardized to the same size for comparison of shape alone. To compare the stress (strength) between models, the models were scaled to the same muscle force: surface area ratio, and to compare strain, the models were scaled to have equal force: volume ratios (following ref. ^83^). Von Mises stress, strain energy (an indicator of work efficiency) and mechanical efficiency were analyzed to compare the biomechanical performance between the models.

### Dental microwear texture analyses

Dental microwear replicas of extant (*T. bairdii*, *T. pinchaque*, and *T. terrestris*; n = 55) and extinct (*Tapirus haysii*, *T. lundeliusi*, *T. polkensis*, and *T. veroensis*; n = 51; see supplemental materials for samples sizes per each extant and extinct taxon) New World tapir species were prepared by molding and casting using polyvinylsiloxane dental impression material (President’s Jet regular body, Coltène-Whaledent Corp., Cuyahoga Falls, OH, USA) and Epotek 301 epoxy resin and hardener (Epoxy Technologies Corp., Billerica, MA, USA), respectively. Extant faunal specimens were accessed in collections housed in the American Museum of Natural History (AMNH; New York City, NY, USA), the Field Museum of Natural History (FMNH; Chicago, IL, USA), and the Yale Peabody Museum (YPM; New Haven, CT, USA). Extinct faunal specimens were examined from the East Tennessee Museum of Natural History and Gray Fossil Site (ETMNH; Johnson City, TN, USA), the Florida Museum of Natural History (UF; Gainesville, FL, USA), and the Texas Memorial Museum (TMM; Austin, TX, USA). Dental microwear texture analysis (DMTA) using white-light confocal profilometry and scale-sensitive fractal analysis (SSFA), was performed on all replicas of bilophodont teeth that preserved ante-mortem microwear similar to prior work (e.g.^37,38,66,67,84–88^.

All specimens were scanned in three dimensions in four adjacent fields of view, for a total sampled area of 204 × 276 µm^2^ and subsequently analyzed using SSFA software (ToothFrax and SFrax, Surfract Corp., www.surfrait.com) to characterize tooth surfaces according to the variables of anisotropy (*epLsar*), complexity (*Asfc*), heterogeneity of complexity (*HAsfc*), and textural fill volume (*Tfv*). Anisotropy is the degree to which surfaces show a preferred orientation, such as the dominance of parallel striations having more anisotropic surfaces (as is typical in folivores and grazers)^66–68,86,89–91,95^. Complexity is the change in surface roughness with scale and used to distinguish taxa that consume hard, brittle foods from those that eat softer/tougher ones^66–68,84,89,95^. Heterogeneity (*HAsfc*_3x3_ and *HAsfc*_9x9_), the degree of texture complexity variation, is measured by calculating *Asfc* variation among subdivided samples (a 3 × 3 and 9 × 9 grid, totaling 9 to 81 subsamples, respectively)^66,67^. Thus, surfaces with high heterogeneity have greater disparity in complexity values between subdivided samples. Lastly, textural fill volume (*Tfv*) measures the volume filled by large (10 µm diameter) and small (2 µm diameter) square cuboids, with high *Tfy* values indicating potentially deeper and/or larger features^38,67,87^.

All statistical analyses follow the same methods of prior DMTA analyses [e.g., 38,86,87]. As dental microwear texture analysis variables are typically non-normally distributed (Shapiro-Wilk tests, p > 0.05), we used non-parametric statistical tests (Kruskal-Wallis and Dunn’s procedure) to conduct multiple comparisons between all extant taxa, and all taxa (extant and extinct) absent of the Bonferroni correction (as the Bonferroni correction increases the probability of false negatives, Type II errors)^92–94^.

### Ethics and data accessibility

All specimens examined were from publically accessible collections as described in the materials and methods section. No permits or permissions were needed to examine these previously collected museum specimens. All primary data are included in the referenced supplemental files.

## Supplementary information

Supplementary information.
