# Supplementary material for: Clarifying relationships between cranial form and function in tapirs, with implications for the dietary ecology of early hominins
Source: Sci Rep. 2020 Jun 1;10:8809. doi: 10.1038/s41598-020-65586-w (PMC7264299; doi:10.1038/s41598-020-65586-w)
Supplement: Supplementary file 1 — Supplementary information. [file 41598_2020_65586_MOESM1_ESM.pdf]

Clarifying relationships between cranial form and function in tapirs with implications for the dietary ecology of early hominins

Larisa R. G. DeSantis[1,2*], Alana C. Sharp[3,4], Blaine W. Schubert[5], Matthew W. Colbert[6], Steven C. Wallace[5], Frederick E. Grine[7]

[1]Department of Earth and Environmental Sciences, Vanderbilt University, Nashville, Tennessee 37235-1805, USA.

[2]Department of Biological Sciences, Vanderbilt University, Nashville, Tennessee 37235-1634, USA.

[3] Institute of Ageing and Chronic Disease, University of Liverpool, Liverpool L69 3BX, United Kingdom

[4] School of Science and Technology, University of New England, Armidale, New South Wales 2351, Australia.

[5]Department of Geosciences and Don Sundquist Center of Excellence in Paleontology, East Tennessee State University, Johnson City, Tennessee 37614, USA

[6]Jackson School of Geosciences, The University of Texas at Austin, Geological Sciences Department, 1 University Station C1100, Austin, Texas 78712, USA

[7]Department of Anthropology, Stony Brook University, Stony Brook, New York 11794, USA

[*]Correspondence: larisa.desantis@vanderbilt.edu

Supplemental Methods and Results

Supplemental Figure 1

Supplemental Tables 1-7

Supplemental Methods

Finite Element Model Construction

All CT data was collected from medical CT scanners and exported in DICOM format. The resulting images were imported into Avizo v9.0 (Visage Imaging, Inc.), where the cranium and mandible were selected using a combination of automated thresholding based on gray values, and they were manually edited to separate the cranium from the mandible and generate 3D surface models. The surface reconstructions were then smoothed and edited to improve the quality of the mesh, including testing the aspect ratio and the dihedral angles of the surface triangles to minimize distorted elements which can cause errors. The aspect ratios of the triangles were adjusted to below 10 (aiming for around 5) and the dihedral angles were set at above 10 degrees to ensure a good quality mesh. The surface meshes were then converted to solid 3D finite element meshes composed of 4-node tetrahedral elements above 1 million elements (supplemental table 2). Finally, each model was exported as an Abaqus input file (*.inp) for easy importation to the FEA software package Abaqus CAE v6.14 (Simulia). The cranium and mandible were imported separately for each species, so that the mandible could be used for muscle force alignment, but excluded from the analysis.

The jaw adductor musculature was modeled as three main components; the temporalis, masseter and pterygoid muscles. Given that there are no measured recordings of bite force or muscle force in tapirs from dissections or *in vivo* experiments, muscle input forces were calculated by multiplying the physiological cross-sectional area (PCSA), estimated using the dry skull method, by 0.3 N, the maximum force of contraction for mammalian muscle fiber [1]. The dry skull method estimates the PCSA of the combined masseter and pterygoid system, so approximately 10-15% of the total muscle force was assigned to the pterygoid for each species. Muscle forces were applied to the skulls by distributing the load for each muscle over the entire surface of the muscle origin. Muscle force orientations were determined by creating a local coordinate system with a vector between the origin and the corresponding insertion on the mandible. A coupling constrain was used to represent the fan shaped nature of the muscle fibers of the temporalis.

Each model was then imported to Abaqus CAE v6.14 where material properties and boundary conditions were applied. To prevent the models from rigid body motion, a single node was constrained at both temporomandibular joints (TMJ) and at each premolar (P2-P4) or molar (M1-M3) on the left, right or both sides for unilateral or bilateral biting, respectively. The left TMJ was fully constrained against displacement in the x- (lateral), y- (vertical), and z-direction (anterior-posterior), and the node on the right TMJ was constrained in the y- and z- axis to allow lateral displacement of the skull. The bite point(s) were constrained in the axis perpendicular to the occlusal plane, so as not to over constrain the model. A number of biting scenarios were modeled to simulate bilateral biting at each individual tooth along the tooth row, and unilateral biting at each tooth with the balancing side muscles adjusted to 60% of the working side. Both left and right unilateral biting was modelled. Even though single node constraints can create artificially high stress and strain in the elements surrounding the constraint, overall patterns of stress and strain distribution are not affected [2,3]. As our goal was to examine overall stress and strain distributions in the skull, with a focus on the function of the sagittal crest not the region around the constraints, these single node constraints are acceptable and minimize the complexity of the model. However, to account for artifacts at these constraints, we examined both the maximum stress and an adjusted maximum in which we removed the top 2% of stress values.

In the absence of material properties data for tapirs, and due to incorporating a fossil specimen with which material properties cannot be estimated from the CT scans, each model was assigned homogeneous and isotropic average values of Young's modulus ($E = 20$ GPa) and Poisson's ratio ($\nu = 0.3$) for mammalian bone [4]. This may produce stiffer models than models with multiple material properties, however, this methodology was considered suitable for the present study, which compares only relative stress and strain values.

Due to the variation in size among the tapir species, the models were standardized to the same size for comparison of shape alone. Stress and strain will depend on the size of the structure. For example, a smaller structure will be stiffer than a larger one, whereas a larger structure will be stronger. All analyses were performed on the original size models and these results were scaled so that the surface

area or volume of each model was equivalent to *T. terrestris*. To compare the stress (strength) between models, the models were scaled to the same ratio of muscle force to surface area. When comparing strain energy, which is the amount of energy stored per unit volume, the models were scaled to the same force: volume ratio, following Dumont and colleagues [5].

Enamel Thickness Estimates.

Avizo was used to non-orthogonally reslice teeth along the protoloph and metaloph orientations (note, protoloph and metalophs were not parallel); measurements were taken on the resliced scans. All measurements were taken on the left upper second molar (M2), except for *T. pinchaque*, where the right M2 was measured (the left being damaged). Measurements were taken on the protoloph and the metaloph (see supplemental figure 1). As CT data for *T. polkensis* showed no contrast between dentine and enamel, and also represented an old individual with visibly worn teeth, it was not measured. All other specimens had the M3 in, or partially in a crypt (the reason for measuring the M2). Since estimating enamel thickness was not a primary aim of our study we did not collect microCT data of the teeth in addition to the lower resolution CT scans used for FEA, therefore these measurements should only be considered estimates. Enamel thickness is reported as raw values, and two metrics were used to remove the effect of body size (as estimated from the width of the protoloph and metaloph of M2s). Specifically, enamel thickness on the protoloph and metaloph were divided by the width of the protoloph or metaloph, respectively. Additionally, enamel thickness was scaled to the size of the largest tooth width (*T. terrestris* in both cases) by dividing smaller teeth by the tooth width of *T. terrestris*, subsequently multiplying the product by the raw enamel thickness values.

Extended Results

Finite element analysis

Unilateral biting. The performance for each model at each bite location for unilateral biting is presented in supplemental table 2. For unilateral biting the mean stress, adjusted maximum stress, and strain energy

for each model decreased along the tooth row from anterior to posterior until approximately half way at which point it increased for all models. *Tapirus bairdii*, however, increased along the entire tooth row for stress and strain, indicating *T. bairdii* is stronger and more energy efficient at more anterior bite points. The highest stress was recorded in *T. pinchaque* for the most anterior bite points, but was higher in *T. bairdii* for the posterior bite points. Mechanical efficiency increased for all models as the bite point moved posteriorly. *T. bairdii* has the highest mechanical efficiency and *T. pinchaque* the lowest.

Bilateral biting. The performance of each model at each bite location for bilateral biting is presented in supplemental table S3. Variation between unilateral and bilateral biting was most notable when comparing stress. For bilateral biting the highest stress was recorded in *T. polkensis* and the lowest was recorded in *T. bairdii* for adjusted maximum and mean stress, but was lowest in *T. terrestris* for maximum stress. However, for unilateral biting *T. polkensis* has the lowest adjusted maximum stress. Strain energy did not vary much between bilateral and unilateral biting and neither did mechanical efficiency.

Enamel Thickness Estimates. Enamel thickness is similar among tapirs (supplemental table 1); however, the largest tapir (*T. bairdii*) has the thinnest enamel, the intermediate sized tapir with the pronounced sagittal crest (*T. terrestris*) has enamel thickness intermediate between *T. bairdii* and *T. pinchaque* (the latter being the smallest extant tapir examined with a reduced sagittal crest). Thus, there does not appear to be a relationship between enamel thickness and either sagittal crest morphology or diet.

References

1. Thomason JJ. 1991 Cranial strength in relation to estimated biting forces in some mammals. *Can. J. Zool.* **69**, 2326-2333.

2. Figueirido B, Tseng ZJ, Serrano-Alarcón FJ, Martín-Serra A, Pastor JF. 2014 Three-dimensional computer simulations of feeding behaviour in red and giant pandas relate skull biomechanics with dietary niche partitioning. *Biol. Letters* **10**, 2014.0196.

3. Dumont ER, Piccirillo J, Grosse IR. 2005 Finite-element analysis of biting behavior and bone stress in the facial skeletons of bats. *Anat. Rec. A. Discov. Mol. Cell. Evol. Biol.* **283** (2), 319-330.

4. Erickson GM, Catanese J, Keaveny TM. 2002 Evolution of the biomechanical material properties of the femur. *Anat. Rec.* **268**, 115-124.

5. Dumont ER, Grosse IR, Slater GJ. 2009 Requirements for comparing the performance of finite element models of biological structures. *J. Theor. Biol.* **256**, 96-103.

Supplemental Figure 1. Images noting the location of enamel thickness measurements of the metaloph (A) and protoloph (B).

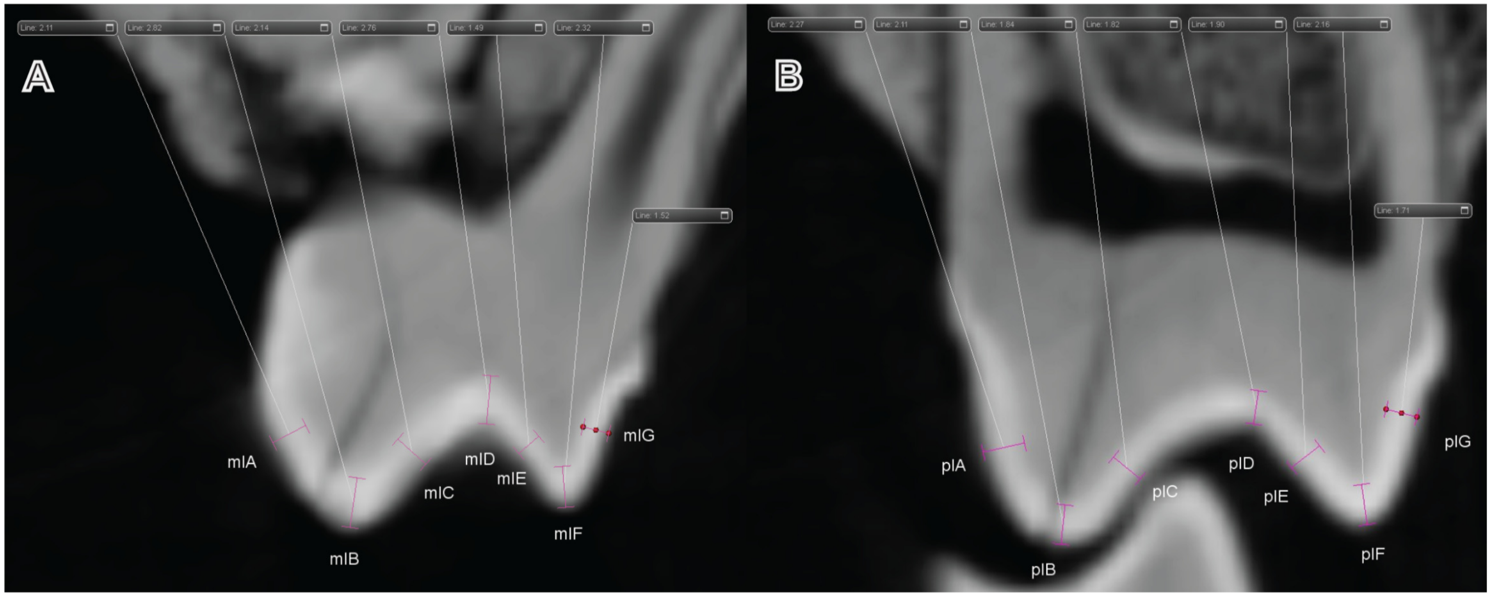

Supplemental Table 1. Enamel thickness measurements from extant tapir CT data.

Taxon and measurement	Width	lA	lB	lC	lD	lE	lF	lG	Average
T. bairdii (ml-raw)	19.28	1.77	2.1	1.66	1.6	1.03	1.6	1.29	1.58
T. bairdii (pl-raw)	23.95	2.04	2.01	1.18	1.54	1.2	1.92	1.49	1.63
T. pinchaque (ml-raw)	20.28	2.07	3.08	1.87	2.05	1.33	2.66	1.59	2.09
T. pinchaque (pl-raw)	24.55	2.13	3.44	2.47	1.99	1.55	3.27	2.28	2.45
T. terrestris (ml-raw)	21.09	2.11	2.82	2.14	2.76	1.49	2.32	1.52	2.17
T. terrestris (pl-raw)	25.32	2.27	2.11	1.84	1.82	1.9	2.16	1.71	1.97
T. bairdii (ml thickness/ml width)	19.28	0.092	0.109	0.086	0.083	0.053	0.083	0.067	0.082
T. bairdii (pl thickness/pl width)	23.95	0.085	0.084	0.049	0.064	0.050	0.080	0.062	0.068
T. pinchaque (ml thickness/ml width)	20.28	0.102	0.152	0.092	0.101	0.066	0.131	0.078	0.103
T. pinchaque (pl thickness/pl width)	24.55	0.087	0.140	0.101	0.081	0.063	0.133	0.093	0.100
T. terrestris (ml thickness/ml width)	21.09	0.100	0.134	0.101	0.131	0.071	0.110	0.072	0.103
T. terrestris (pl thickness/pl width)	25.32	0.090	0.083	0.073	0.072	0.075	0.085	0.068	0.078
T. bairdii (thickness*(T.t. ml width/T.b. ml width))	19.28	1.94	2.30	1.82	1.75	1.13	1.75	1.41	1.73
T. bairdii (thickness*(T.t. pl width/T.b. pl width))	23.95	2.16	2.12	1.25	1.63	1.27	2.03	1.58	1.72
T. pinchaque (thickness*(T.t. ml width/T.p. ml width))	20.28	2.15	3.20	1.94	2.13	1.38	2.77	1.65	2.18
T. pinchaque (thickness*(T.t. pl width/T.p. pl width))	24.55	2.20	3.55	2.55	2.05	1.60	3.37	2.35	2.52
T. terrestris (thickness*(T.t. ml width/T.b. ml width))	21.09	2.11	2.82	2.14	2.76	1.49	2.32	1.52	2.17
T. terrestris (thickness*(T.t. pl width/T.b. pl width))	25.32	2.27	2.11	1.84	1.82	1.90	2.16	1.71	1.97

Width, width in mm of the metaloph and protoloph of the second upper molar which provide a proxy of body size; lA-G, measurements corresponding to mlA-G and plA-G in Supplemental Figure 1 (all noted in mm); Average, average of all lA-G measurements per either the metaloph or protoloph; T.t., *Tapirus terrestris*, T.b., *Tapirus bairidii*, T.p. *Tapirus pinchaque*.

Supplemental Table 2. Properties for each finite element model.

	T. terrestris	*T. bairdii*	*T. pinchaque*	*T. polkensis*
Mean element edge length	1.65	1.32	1.34	1.31
Number of elements	2,193,829	1,668,252	1,376,868	1,770,918

Supplemental Table 3. Performance metrics for unilateral biting at each bite point (1-6, corresponding to the upper P2, P3, P4, M1, M2, and M3) along the tooth row from anterior to posterior on the left side. All stress values are in megapascals and all strain values are in microjouls. Adjusted maximum stress is the maximum stress with the top 2% of values removed to account of modeling artefacts from single node constraints.

Bite Point	Variables	*T. terrestris*	*T. bairdii*	*T. pinchaque*	*T. polkensis*
1	Maximum stress	387	505	545	268
	Mean stress	1.38	1.00	1.51	1.39
	Adjusted max stress	6.28	5.45	6.41	5.42
	Strain energy	231	123	155	125
	Mechanical efficiency	0.23	0.23	0.18	0.20
2	Maximum stress	298	518	296	426
	Mean stress	1.30	1.21	1.42	1.29
	Adjusted max stress	5.80	5.25	5.95	4.89
	Strain energy	210	106	135	125
	Mechanical efficiency	0.25	0.25	0.20	0.22
3	Maximum stress	231	527	600	287
	Mean stress	1.25	1.21	1.36	1.23
	Adjusted max stress	5.58	5.36	5.70	4.42
	Strain energy	196	118	136	107
	Mechanical efficiency	0.27	0.28	0.22	0.24
4	Maximum stress	282	517	284	597
	Mean stress	1.21	1.25	1.31	1.21
	Adjusted max stress	5.35	5.72	5.48	4.25
	Strain energy	205	132	123	124
	Mechanical efficiency	0.31	0.32	0.25	0.27
5	Maximum stress	529	471	652	832
	Mean stress	1.20	1.35	1.32	1.24
	Adjusted max stress	5.51	6.24	5.67	4.50
	Strain energy	219	154	139	146
	Mechanical efficiency	0.36	0.38	0.30	0.32
6	Maximum stress	311	627	443	578
	Mean stress	1.29	1.64	1.50	1.42
	Adjusted max stress	6.29	8.19	7.03	5.58
	Strain energy	257	271	175	181
	Mechanical efficiency	0.43	0.48	0.38	0.40

Supplemental Table 4. Performance metrics for bilateral biting at each bite point (1-6, corresponding to the upper P2, P3, P4, M1, M2, and M3) along the tooth row from anterior to posterior on the left side. All stress values are in megapascals and all strain values are in microjouls. Adjusted maximum stress is the maximum stress with the top 2% of values removed to account of modeling artefacts from single node constraints.

Bite Point	Variables	*T. terrestris*	*T. bairdii*	*T. pinchaque*	*T. polkensis*
1	Maximum stress	302	399	334	523
	Mean stress	1.57	1.22	1.76	2.27
	Adjusted max stress	6.73	4.83	7.24	8.45
	Strain energy	282	114	169	134
	Mechanical efficiency	0.28	0.28	0.22	0.24
2	Maximum stress	292	394	332	512
	Mean stress	1.47	1.16	1.64	2.07
	Adjusted max stress	6.24	4.44	6.77	7.54
	Strain energy	254	98	154	124
	Mechanical efficiency	0.30	0.31	0.25	0.26
3	Maximum stress	277	380	333	484
	Mean stress	1.40	1.11	1.56	1.92
	Adjusted max stress	5.79	4.25	6.33	6.71
	Strain energy	232	95	141	105
	Mechanical efficiency	0.34	0.35	0.28	0.30
4	Maximum stress	263	362	295	487
	Mean stress	1.32	1.08	1.48	1.82
	Adjusted max stress	5.20	4.21	5.83	6.25
	Strain energy	214	94	126	99
	Mechanical efficiency	0.38	0.40	0.31	0.34
5	Maximum stress	343	316	463	675
	Mean stress	1.24	1.03	1.38	1.70
	Adjusted max stress	4.83	4.06	5.47	6.03
	Strain energy	210	93	122	98
	Mechanical efficiency	0.44	0.47	0.37	0.40
6	Maximum stress	446	332	316	479
	Mean stress	1.19	1.07	1.34	1.65
	Adjusted max stress	4.96	4.72	5.81	6.18
	Strain energy	209	132	117	103
	Mechanical efficiency	0.53	0.59	0.47	0.49

Supplemental Table 5. Descriptive statistics for each DMTA variable for each extant and extinct taxon.

Taxon	Statistic	*n*	*Asfc*	*epLsar*	*Tfv*	$HAsfc_{(3x3)}$	$HAsfc_{(9x9)}$
Tapirus bairdii	Median	24	2.132	0.0025	11717.831	0.454	0.870
(extant)	Mean		4.285	0.0034	10440.871	0.509	0.903
	Standard Deviation		4.888	0.0024	5367.977	0.211	0.330
	Minimum		0.796	0.0008	605.850	0.219	0.436
	Maximum		19.409	0.0099	18379.212	1.017	1.604
	Total Range		18.613	0.0091	17773.362	0.798	1.167
	Skewness (Fisher)		2.001	1.602	-0.481	0.874	0.399
	p for normality (Shapiro-Wilk)		<0.0001*	0.001*	0.159	0.114	0.395
Tapirus pinchaque	Median	7	0.858	0.0043	5613.120	0.369	0.616
(extant)	Mean		0.793	0.0049	7000.464	0.363	0.678
	Standard Deviation		0.302	0.0016	3806.802	0.100	0.196
	Minimum		0.250	0.0035	1881.665	0.258	0.451
	Maximum		1.231	0.0078	11904.615	0.560	0.996
	Total Range		0.981	0.0042	10022.950	0.302	0.545
	Skewness (Fisher)		-0.674	1.181	0.127	1.307	0.796
	p for normality (Shapiro-Wilk)		0.560	0.099	0.568	0.141	0.361
Tapirus terrestris	Median	24	1.389	0.0032	5829.912	0.448	0.775
(extant)	Mean		1.894	0.0036	6021.906	0.505	0.869
	Standard Deviation		1.425	0.0016	3927.095	0.242	0.374
	Minimum		0.435	0.0018	0.000	0.190	0.359
	Maximum		5.914	0.0080	12890.398	1.087	1.695
	Total Range		5.478	0.0062	12890.398	0.897	1.336
	Skewness (Fisher)		1.852	1.017	0.207	0.648	0.651
	p for normality (Shapiro-Wilk)		<0.001*	0.024*	0.394	0.048*	0.114
Tapirus haysii†	Median	8	2.347	0.0024	11687.287	0.639	1.193
(extinct)	Mean		3.910	0.0025	11071.566	0.639	1.121
	Standard Deviation		3.042	0.0012	3914.953	0.175	0.259
	Minimum		1.426	0.0010	3721.587	0.347	0.576
	Maximum		9.982	0.0043	14944.162	0.884	1.342
	Total Range		8.556	0.0033	11222.574	0.537	0.766
	Skewness (Fisher)		1.324	0.574	-0.901	-0.259	-1.493
	p for normality (Shapiro-Wilk)		0.042*	0.405	0.333	0.930	0.070
Tapirus lundeliusi†	Median	9	1.655	0.0019	6568.437	0.522	1.019
(extinct)	Mean		2.468	0.0020	6368.786	0.671	1.419
	Standard Deviation		2.461	0.0006	4915.762	0.332	0.900
	Minimum		0.696	0.0010	505.078	0.327	0.555
	Maximum		8.323	0.0031	15202.840	1.327	3.307
	Total Range		7.628	0.0021	14697.763	1.000	2.753
	Skewness (Fisher)		2.105	0.291	0.327	1.040	1.368
	p for normality (Shapiro-Wilk)		0.001*	0.839	0.422	0.210	0.036*
Tapirus polkensis†	Median	20	0.938	0.0035	8790.869	0.427	0.835

(extinct)	Mean		1.080	0.0042	6879.231	0.468	0.864
	Standard Deviation		0.608	0.0020	4273.964	0.199	0.344
	Minimum		0.440	0.0015	0.000	0.204	0.354
	Maximum		2.816	0.0085	12909.356	0.949	1.747
	Total Range		2.375	0.0070	12909.356	0.745	1.393
	Skewness (Fisher)		1.244	0.729	-0.560	0.932	0.834
	p for normality (Shapiro-Wilk)		0.019*	0.098	0.011*	0.145	0.317
Tapirus veroensis† (extinct)	Median	14	3.691	0.0023	13052.230	0.480	0.817
	Mean		4.650	0.0024	12419.386	0.535	0.892
	Standard Deviation		3.073	0.0009	3490.060	0.208	0.329
	Minimum		1.559	0.0011	3641.173	0.262	0.492
	Maximum		10.441	0.0040	17189.742	0.858	1.488
	Total Range		8.882	0.0029	13548.569	0.596	0.996
	Skewness (Fisher)		1.238	0.518	-1.105	0.421	0.482
	p for normality (Shapiro-Wilk)		0.003*	0.432	0.287	0.116	0.111

n, number of individuals sampled*;* *Asfc*, area-scale fractal complexity; *epLsar*, anisotropy; *Tfv*, textural fill volume; $HAsfc_{3x3}$, $HAsfc_{9x9}$, Heterogeneity of complexity in a 3x3 and 9x9 grid, respectively.
* = denotes significant p-values ($p<0.05$).

Supplemental Table 6. Statistical results for taxonomic comparisons of complexity (*Asfc*), anisotropy (*epLsar*), and textural fill volume (*Tfv*) values for all extant and extinct tapirs here examined.

Variable		*T. pinchaque*	*T. terrestris*	*T. haysii*†	*T. polkensis*†	*T. lundeliusi*†	*T. veroensis*†
complexity (*Asfc*)	*T. bairdii*	**<0.001**	**0.036**	0.568	**<0.0001**	0.236	0.106
	T. pinchaque		**0.025**	**<0.001**	0.467	**0.029**	**<0.0001**
	T. terrestris			**0.040**	**0.034**	0.718	**<0.001**
	T. haysii†				**<0.001**	0.152	0.484
	T. polkensis†					0.051	**<0.0001**
	T. lundeliusi†						**0.018**
anisotropy (*epLsar*)	*T. bairdii*	**0.015**	0.287	0.319	0.073	**0.047**	0.252
	T. pinchaque		0.087	**0.005**	0.254	**<0.001**	**0.002**
	T. terrestris			0.080	0.438	**0.006**	**0.039**
	T. haysii†				**0.023**	**0.001**	0.961
	T. polkensis†					**0.001**	**0.008**
	T. lundeliusi†						0.361
textural fill volume (*Tfv*)	*T. bairdii*	0.096	**0.002**	0.732	**0.015**	**0.033**	0.176
	T. pinchaque		0.646	0.098	0.963	0.818	**0.011**
	T. terrestris			**<0.01**	0.558	0.835	**<0.0001**
	T. haysii†				**0.036**	**0.046**	0.477
	T. polkensis†					0.554	**<0.001**
	T. lundeliusi†						**0.003**

*p-values < 0.05, also in bold. † denotes extinct taxon.

Supplemental Table 7. DMTA data for each extant and extinct taxon here examined.

Status	Taxon	Museum ID	Tooth	*Asfc*	*epLsar*	*Tfv*	$HAsfc_{3x3}$	$HAsfc_{9x9}$
Extant	*Tapirus bairdii*	FMNH14898	RM1	1.548	0.0019	722	0.433	0.854
		FMNH14899	LM1	1.606	0.0043	12294	0.755	1.137
		FMNH15978	RM2	1.878	0.0018	5190	0.759	1.248
		FMNH15979	RM1	2.777	0.0036	10508	0.420	0.965
		FMNH22402	LM2	0.796	0.0091	13013	0.349	0.455
		FMNH34665	LM2	3.495	0.0025	3849	0.475	1.058
		FMNH34666	LM1	1.672	0.0015	9128	0.358	0.555
		YPM6712	LM2	1.457	0.0049	8198	0.233	0.564
		YPM7132	LM2	2.077	0.0027	17700	0.593	0.969
		YPM7133	LM2	12.374	0.0025	18117	0.628	1.093
		YPM7135	LM2	6.733	0.0020	14803	0.593	1.240
		YPM7136	LM2	1.196	0.0099	12480	0.601	0.764
		YPM7140	LM2	1.355	0.0034	1044	0.219	0.449
		YPM7141	LM2	14.365	0.0008	18379	0.392	0.734
		YPM7143	LM2	2.935	0.0037	12019	0.700	1.274
		YPM7475	LM2	0.997	0.0074	12187	0.302	0.512
		YPM7476	LM2	10.233	0.0020	15341	0.498	0.810
		YPM7477	LM2	1.116	0.0036	606	0.400	0.741
		YPM8624	LM1	1.620	0.0022	8440	0.425	1.027
		YPM8627	LM1	2.280	0.0014	11417	0.945	1.604
		YPM8631	LM1	4.964	0.0051	10769	1.017	1.529
		YPM9398	LM2	2.186	0.0025	5167	0.313	0.436
		YPM9746	LM1	19.409	0.0011	15111	0.503	0.886
		YPM9747	LM2	3.775	0.0024	14100	0.312	0.775
	Tapirus pinchaque	AMNH149331	LM2	0.912	0.0035	1882	0.258	0.522
		AMNH149332	LM2	0.886	0.0037	5613	0.369	0.996
		AMNH70521	LM2	1.231	0.0064	3861	0.266	0.451
		FMNH47051	LM2	0.808	0.0078	9167	0.336	0.616
		FMNH70557	RM2	0.608	0.0037	11905	0.560	0.892
		FMNH89207	LM1	0.858	0.0043	11196	0.379	0.616
		FMNH90023	LM2	0.250	0.0049	5381	0.372	0.654
	Tapirus terrestris	AMNH120996	LM3	1.200	0.0042	12890	0.490	0.794
		AMNH142280	RM2	1.242	0.0055	10294	0.278	0.480
		AMNH14690	LM2	1.280	0.0053	4346	0.272	0.587
		AMNH209139	LM2	5.914	0.0021	3415	0.788	1.397
		AMNH217150	LM2	2.080	0.0043	1478	0.368	0.656
		AMNH246974	LM2	5.643	0.0024	11332	0.274	0.756
		AMNH36661	LM2	1.626	0.0080	12231	0.463	0.992
		AMNH73596	RM2	0.808	0.0038	335	0.190	0.392
		AMNH73766	RM2	0.908	0.0035	597	0.307	0.526
		AMNH77573	LM2	0.810	0.0030	8183	0.331	0.557

		AMNH77576	RM2	0.541	0.0057	2943	0.740	1.444
	Tapirus terrestris	AMNH95133	LM2	2.853	0.0034	8371	0.816	0.942
		AMNH96130	LM2	1.310	0.0025	0	0.794	1.198
		AMNH98685	RM2	2.231	0.0020	6147	0.433	0.608
		FMNH20028	RM2	0.435	0.0019	3362	0.389	0.723
		FMNH21377	LM2	2.191	0.0018	9753	0.312	0.543
		FMNH21923	LM2	1.612	0.0053	4778	0.201	0.359
		FMNH28273	LM3	1.201	0.0030	5974	0.670	0.952
		FMNH34263	LM2	0.803	0.0019	4353	0.312	0.603
		FMNH34264	LM2	1.126	0.0043	5686	0.487	0.851
		FMNH34265	LM2	2.473	0.0030	2206	0.692	1.456
		FMNH34268	LM2	1.468	0.0047	6943	0.771	1.216
		FMNH34274	LM1	1.941	0.0023	7202	1.087	1.695
		FMNH34275	RM1	3.763	0.0021	11706	0.650	1.133
Extinct (Miocene/Pliocene)	*Tapirus polkensis*	ETMNH3425	RM2	1.243	0.0030	1441	0.383	0.959
		ETMNH3426	LM2	1.450	0.0032	8917	0.430	0.818
		ETMNH3427	LM2	0.624	0.0040	12909	0.224	0.601
		ETMNH3700*	RM2	1.579	0.0059	976	0.323	0.688
		ETMNH3711	LM2	0.521	0.0070	0	0.286	0.798
		ETMNH3843**	LM2	0.463	0.0033	5790	0.510	1.747
		ETMNH595	RM2	0.440	0.0043	7684	0.534	0.976
		ETMNH602	LM2	1.149	0.0037	8895	0.627	0.974
		ETMNH606*	RM2	1.477	0.0019	9467	0.500	0.852
		ETMNH607*	RM2	0.563	0.0025	2358	0.536	1.085
		ETMNH608	LM2	1.299	0.0032	0	0.395	0.512
		ETMNH611*	RM2	0.897	0.0067	9426	0.949	1.400
		ETMNH639	LM2	0.794	0.0061	7703	0.297	0.505
		ETMNH652	RM2	1.688	0.0028	997	0.700	1.226
		ETMNH661	LM2	0.773	0.0085	9564	0.422	0.539
		ETMNH664	RM3	0.515	0.0065	10127	0.244	0.494
		ETMNH680*	LM2	0.978	0.0040	12321	0.425	0.669
		ETMNH681**	RM2	1.831	0.0018	9657	0.516	1.061
		ETMNH682*	LM2	2.816	0.0032	8686	0.851	1.017
		ETMNH683**	LM2	0.493	0.0015	10666	0.204	0.354
Extinct (Pleistocene)	*Tapirus haysii*	UF65973	rp2	5.445	0.0014	14419	0.631	1.226
		UF85314	lm	9.982	0.0010	14944	0.884	1.320
		UF86702	RP1	6.183	0.0026	11041	0.753	1.342
		UF86851	RM	1.919	0.0043	14721	0.520	0.997
		UF86941	rm2	2.052	0.0022	12333	0.647	1.331
		UF87941	rp3	1.426	0.0026	9357	0.347	0.576
		UF87957	lm	1.634	0.0015	8036	0.806	1.159
		UF89533	RM3	2.642	0.0040	3722	0.523	1.015
	Tapirus lundeliusi	UF221720	lm2	1.277	0.0023	716	0.987	3.307
		UF224670	lm3	1.002	0.0017	9683	0.444	0.944

	UF224672	rp2	4.494	0.0019	9212	0.401	1.037
	UF224674	RM2	1.114	0.0010	505	0.865	1.981
	UF224679	lp4	8.323	0.0019	15203	1.327	2.230
	UF224680	lm2	1.655	0.0031	1277	0.327	0.555
	UF224683	LM	1.921	0.0020	8282	0.522	0.887
	UF244503	LM2	1.729	0.0027	5872	0.707	1.019
	UF244600	lm3	0.696	0.0016	6568	0.456	0.809
Tapirus veroensis	TMM30967-1161	lp4	9.344	0.0022	15798	0.800	1.113
	TMM30967-1211	LM1	2.442	0.0011	8863	0.823	1.488
	TMM30967-1237	lm2	3.758	0.0021	13848	0.366	0.492
	TMM30967-2176	RM2	2.203	0.0033	16352	0.451	0.682
	TMM30967-222	RP	2.446	0.0023	12604	0.508	0.953
	TMM30967-281	lm1	2.782	0.0015	11298	0.288	0.615
	TMM30967-74	RM2	1.559	0.0032	3641	0.262	0.576
	TMM30967-917	rm1	3.803	0.0024	17190	0.345	0.589
	TMM30967-928	RM2	5.132	0.0038	12992	0.434	0.668
	TMM30976-1984	rm2	10.441	0.0022	13112	0.644	1.232
	UF209299	lp4	3.624	0.0040	10080	0.508	0.972
	UF210878	RM3	10.331	0.0017	14530	0.783	1.356
	UF210890	rm3	2.827	0.0026	10345	0.421	0.612
	UF210891	lm3	4.408	0.0015	13217	0.858	1.139

Museum ID: AMNH, American Museum of Natural History; FMNH, Field Museum of Natural History; TMM, Texas Memorial Museum; UF, Florida Museum of Natural History; YPM, Yale Peabody Museum; Tooth, tooth positions (lower case, mandibular teeth; upper case, maxillary teeth, right or left sides are noted along with molar, m, and premolar, p); *Asfc*, area-scale fractal complexity; *epLsar*, anisotropy; *Tfv*, textural fill volume; $HAsfc_{3x3}$, $HAsfc_{9x9}$, heterogeneity of complexity in a 3x3 and 9x9 grid, respectively.
*= low parasagittal ridges that do not unite to form a sagittal crest in adults, or **=sagittal crest present in *T. polkensis* at the Gray Fossil site.
